# Adult IDH-wildtype glioblastoma in Mexico and Latin America: contemporary standards, emerging strategies, and an evidence-to-implementation roadmap

**DOI:** 10.3389/fonc.2026.1998982

**Published:** 2026-10-28

**Authors:** Alejandro Rodríguez Camacho, Emmanuel Polito-Salazar, Ximena Blasco, Juan Carlos Romero-Valencia, Irving Fuentes-Calvo, Dharely Cid-Sánchez, José Guillermo Flores-Vázquez, Monica Serrano-Murillo, Bernardo Cacho-Díaz, Alipio González Vázquez, José Omar Santellán Hernández, Daniel Alejandro Vega Moreno, Enrique Caballé-Pérez, Daniel San-Juan, Emilio García-Gómez, Daniel Ballesteros-Herrera, Talia Wegman-Ostrosky, Oswaldo Alan Chávez Martínez, Efraín Isaías Camarin Sánchez, Raúl Mellado Orellana, Karen Elizabeth Padilla-Leal, Julia Moscardini-Martelli, Andrea Garcia-Bitar, Federico Maldonado Magos, Jorge Alejandro Torres-Ríos, Miguel Ángel Celis López, Guillermo Axayacalt Gutiérrez-Aceves, Sergio Moreno-Jiménez

**Affiliations:** 1 Radioneurosurgery Unit, National Institute of Neurology and Neurosurgery Manuel Velasco Suárez, Mexico, Mexico; 2 Epilepsy Clinic, National Institute of Neurology and Neurosurgery Manuel Velasco Suárez, Mexico, Mexico; 3 Neurosurgery Service, National Institute of Neurology and Neurosurgery Manuel Velasco Suárez, Mexico, Mexico; 4 UMAE Oncology Hospital, Centro Médico Nacional Siglo XXI, Mexican Social Security Institute IMSS, Mexico, Mexico; 5 Neuro-Oncology, Centro Oncológico Fatima, Los Mochis, Sinaloa, Mexico; 6 Functional Unit of Neuro-Oncology, Instituto Nacional de Cancerología, Mexico, Mexico; 7 Oncological Neurosurgery, Ángeles Pedregal Hospital, Mexico, Mexico; 8 Oncology, Ángeles Pedregal Hospital, Mexico, Mexico; 9 Neuro-Oncology, National Institute of Neurology and Neurosurgery Manuel Velasco Suárez, Mexico, Mexico; 10 Oncological Neurosurgery Department, National Institute of Neurology and Neurosurgery Manuel Velasco Suárez, Mexico, Mexico; 11 Department of Neurological Surgery, The American British Cowdray Medical Center, Mexico, Mexico

**Keywords:** glioblastoma, IDH-wildtype, implementation science, Latin America, molecular diagnostics, neurosurgery, radiotherapy

## Abstract

Glioblastoma remains a major therapeutic challenge, and disparities in diagnostic capacity, treatment access, and continuity of care may further limit outcomes in Mexico and Latin America. This structured narrative review examines contemporary management of glioblastoma in adults, focusing on implementation of evidence-based care across resource-variable health systems. We reviewed literature published from 2005 through August 2026, including guidelines, consensus statements, randomized trials, prospective and registry-based studies, systematic reviews, meta-analyses, regional cohorts, epidemiological data, and health-system evidence. Current evidence supports integrated molecular diagnosis, maximal safe resection, postoperative radiotherapy with temozolomide, individualized management at recurrence, and early supportive and palliative care. Molecular classification and standardized imaging are essential to treatment selection and response assessment, whereas emerging targeted, immune, cellular, DNA-damage response, blood–brain barrier, and artificial intelligence approaches remain dependent on indication-specific evidence and appropriate clinical-trial or specialist oversight. Contemporary epidemiological and real-world studies provide external survival benchmarks, although differences in molecular classification, population selection, and study design limit direct comparisons with regional estimates and clinical-trial outcomes. Regional studies identify gaps in molecular testing, radiotherapy access, treatment continuity, and socioeconomic and geographic equity; however, evidence remains concentrated in a limited number of countries and tertiary centers, limiting its representativeness across Latin America. We propose a conceptual, resource-stratified, bidirectional local-to-hub framework in which routine care remains local when quality standards can be met, while specialist centers provide complex diagnostics, procedures, treatment review, recurrence assessment, and research access. Shared infrastructure for remote review, specimen and data transfer, patient navigation, and prospective quality measurement may reduce avoidable barriers without lowering clinical standards. The framework distinguishes established care from specialist-supported and investigational strategies and proposes indicators to evaluate diagnostic timeliness, treatment completion, referral performance, supportive care, equity, and research access. Improving outcomes requires not only access to effective therapies, but reliable systems capable of delivering them consistently and equitably throughout the course of disease.

## 1 Introduction

Glioblastoma (GBM) remains one of the most challenging malignancies in neuro-oncology. Maximal safe resection followed by radiotherapy and temozolomide has improved survival, but median overall survival remains approximately 15–18 months, and durable long-term survival is uncommon (1–3). The 2021 WHO Classification of CNS Tumors (WHO CNS5) integrates histological and molecular features and reserves the term GBM for IDH-wildtype tumors. IDH-mutant grade 4 astrocytic tumors are classified as astrocytoma, IDH-mutant, CNS WHO grade 4 (4, 5). Nevertheless, tumor heterogeneity, cellular plasticity, diffuse infiltration, and limited drug delivery continue to constrain durable therapeutic benefit (6–9).

The burden of GBM in Mexico and Latin America is difficult to quantify because available cancer registries rarely capture the disease at molecular resolution. GLOBOCAN 2024 estimated 27,774 new malignant brain and central nervous system cancer cases, 23,229 deaths, and 98,201 five-year prevalent cases in Latin America and the Caribbean; corresponding estimates for Mexico were 3,039 new cases, 2,583 deaths, and 10,751 five-year prevalent cases (10). Regional studies from Brazil, Argentina, Mexico, and Chile describe disparities in diagnosis, access, and outcomes, although their populations and study designs are not directly comparable (11–17). In Mexico, geographic access to radiotherapy represents an additional constraint: 23.38% of the population, approximately 29.5 million people, lives at least 100 km from the nearest radiotherapy facility (15).

Studies of high-grade and mixed glioma populations in low- and middle-income countries have reported limited surgical access, incomplete adjuvant treatment, restricted molecular characterization, treatment abandonment, and loss to follow-up (18, 19). These gaps have been described as sequential “survival gates.” A GBM-specific meta-analysis found longer survival in studies conducted after the adoption of chemoradiotherapy, without a significant difference according to the World Bank income category (20). Another meta-analysis found poorer survival among patients living in economically disadvantaged settings (21). These findings support closer examination of how care is delivered and completed, but they do not establish a causal effect for any individual barrier.

This structured narrative review examines how contemporary GBM evidence can be applied within health systems with differing diagnostic, treatment, and research capacity. We address established management, emerging therapies, and the practical requirements for their use in Mexico and Latin America, recognizing substantial heterogeneity across countries and populations. The regional framework is intended as an implementation context rather than as a claim of epidemiological or biological representativeness, with country-specific evidence interpreted within its local context. The framework retains a common clinical standard while allowing service development and referral pathways to reflect local capacity. Its purpose is to identify gaps that can be addressed locally, define when specialist referral is needed, and establish measures that can be evaluated over time.

## 2 Methods

This structured narrative review examined contemporary evidence on GBM in adults, with particular attention to its implementation in Mexico and Latin America. We searched PubMed/MEDLINE, Scopus, Web of Science, GLOBOCAN, major society guidelines, consensus statements, and regional policy and health-system capacity reports for English- and Spanish-language literature published from January 1, 2005, through August 19, 2026. The search used combinations of terms related to GBM, IDH-wildtype, WHO CNS5, molecular diagnosis, IDH1, IDH2, MGMT, surgery, radiotherapy, temozolomide, recurrent GBM, supportive care, artificial intelligence, health systems, access, implementation, Mexico, and Latin America, together with modality- and treatment-specific terms as appropriate.

Studies were prioritized according to their relevance to GBM and to contemporary clinical practice or implementation. Randomized clinical trials, current guidelines, prospective and registry-based studies, systematic reviews and meta-analysis, contemporary epidemiological studies, and regional evidence addressing diagnosis, treatment delivery, outcomes, access, or health-system capacity were given priority. Early-phase and translational studies were included when they provided relevant information on biological rationale, safety, pharmacology, feasibility, or preliminary activity, but were not considered practice-changing without confirmatory evidence. Studies with limited direct relevance to GBM were used selectively when necessary to provide biological, historical, or contextual background.

Regional evidence was identified by combining disease- and treatment-related search terms with geographic terms and was selected when it provided information relevant to Mexico or other Latin American health systems, including epidemiology, molecular diagnostic capacity, treatment access and delivery, outcomes, resource constraints, or implementation. Latin America was considered as a regional implementation context rather than a homogeneous epidemiological or biological population; country-specific evidence was therefore interpreted within its local context and was not assumed to be representative of the region as a whole. Because this was a structured narrative review rather than a systematic review, study selection was based on clinical relevance, methodological strength, contemporary applicability, and relevance to the regional implementation context rather than formal duplicate screening or predefined quantitative eligibility criteria. We therefore did not perform duplicate screening, formal risk-of-bias assessment, or quantitative synthesis. The complete search strategy, including the main search terms, is detailed in **Supplementary Material 1** to enhance methodological transparency and reproducibility.

## 3 Clinical framework: care delivery and capability stratification

GBM care depends on linked clinical functions, from obtaining diagnostic tissue and completing molecular characterization to planning treatment, assessing response, and managing recurrence. International recommendations define these functions, but their availability and coordination vary across health systems (3, 5, 22, 23). Regional studies document gaps in molecular testing, geographic access, and continuity of care that can disrupt this pathway (18–21).

Resource stratification should therefore determine how care is delivered, rather than establish different clinical standards. A center may meet an essential requirement through reliable referral without providing every service on site. We distinguish universal standards, referral-hub capabilities, audited pilots, and trial- or registry-only strategies according to the evidence, expertise, infrastructure, and quality assurance required for their delivery. These categories describe service organization, not different treatment standards for patients.

Referral pathways should allow specialist review of imaging, pathology, and treatment plans when travel is unnecessary, while procedures requiring in-person care should include a clear plan for onward management. **Figure 1** illustrates this bidirectional local-to-hub model, while **Table 1** summarizes the international standards and regional evidence supporting its implementation.

**Figure 1:**
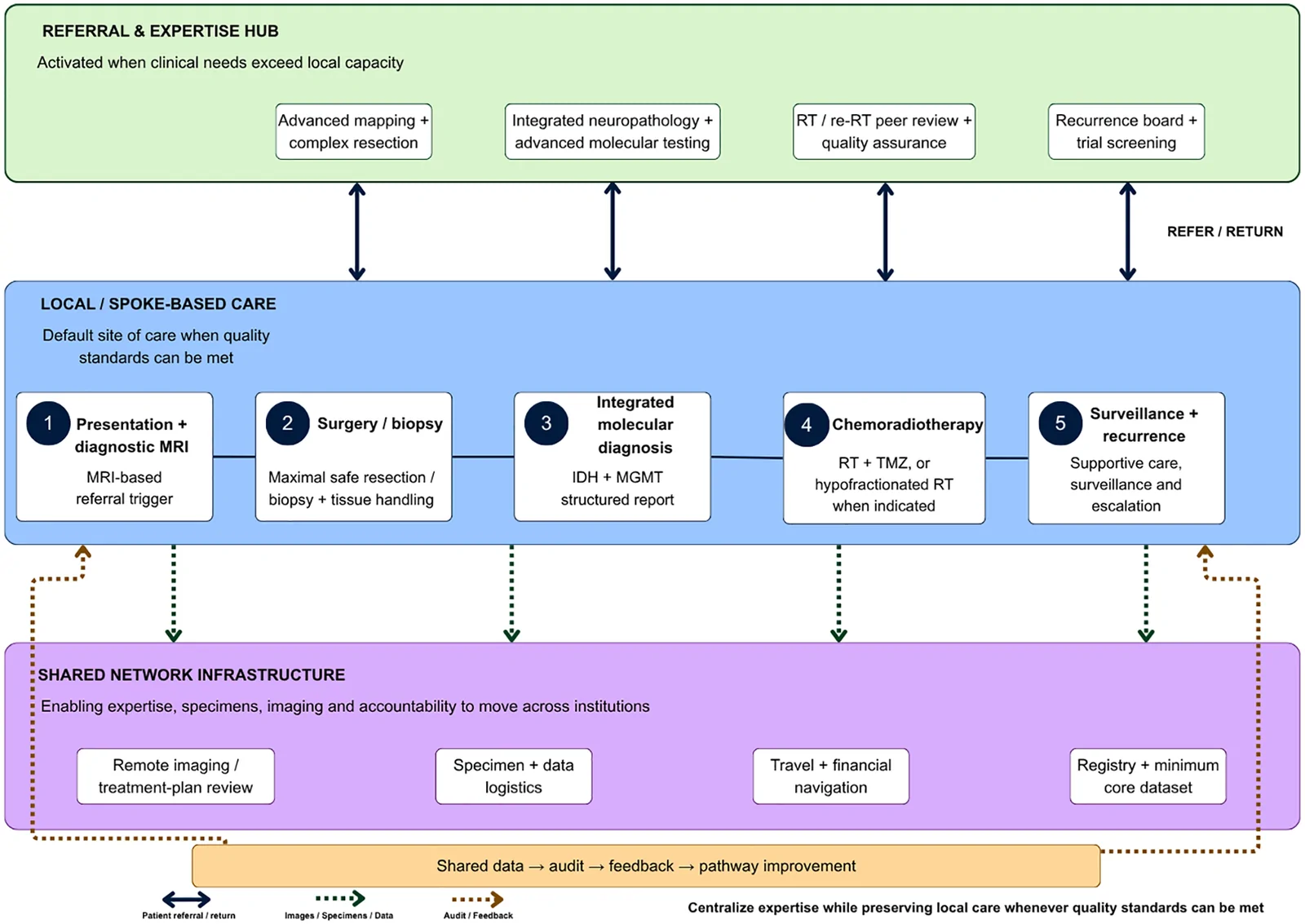
Resource-stratified, bidirectional local-to-hub care pathway for glioblastoma. Local or spoke-based care remains the default when core quality standards can be met, while referral hubs provide specialized expertise and infrastructure for complex clinical and diagnostic needs. Patients may return to the closest qualified local service after specialist assessment or intervention. Shared infrastructure supports remote review, data and specimen transfer, travel navigation, and audit. Solid arrows represent patient referral and return; dashed arrows represent information and specimen transfer; dotted arrows represent audit and feedback.

**Table 1:** International reference standards and the regional evidence informing this review.

International reference standards and the regional evidence informing this review		
Care domain	International reference standard	Regional evidence and limits of inference
Integrated diagnosis	WHO CNS5 and the EANO molecular-diagnostic guideline define the histomolecular diagnosis and the tests required in the relevant setting; MGMT informs treatment selection (5, 27).	Brazilian multicenter and Argentine tertiary-center studies document incomplete molecular assessment. Both include broader glioma populations; neither estimates access for every Latin American health system (16, 17).
Imaging and response assessment	BTIP supports comparable MRI acquisition; RANO 2.0 and PET-RANO define response-assessment approaches. RANO-Resect standardizes residual-volume reporting (40–43).	A Mexico-focused clinical perspective identifies implementation needs (13). Region-wide rates of protocol completion, expert review, or tracer access are not established by the sources included here.
Surgery	The SNO/EANO review supports maximal safe resection or biopsy according to patient condition and functional risk; resection studies must be interpreted with selection effects (3, 41, 45).	The Mexican perspective and Chilean outcome series describe particular clinical settings. They cannot establish regional availability of mapping, intraoperative imaging, or specialist reoperation (12, 13).
Radiotherapy and systemic treatment	EORTC/NCIC evidence, ASTRO, and ESTRO–EANO support indicated chemoradiotherapy and appropriate shorter-course pathways for older or frail patients (1, 22, 23, 28, 53).	The Mexican geospatial study measures distance to radiotherapy facilities, not GBM treatment completion. Brazilian socioeconomic data describe associations with outcomes rather than a causal estimate of each access barrier (11, 15).
Recurrent disease	SNO/EANO and ESTRO/EANO guide individualized reassessment and selected local treatment. RTOG 1205 demonstrated a PFS benefit, without an OS benefit, for its combined reirradiation regimen (3, 54, 55).	The included regional literature does not quantify access to every salvage option. Referral and treatment pathways in this review are proposals for local evaluation, not a country-level availability survey.
Supportive care and equity	EANO palliative-care, SNO/EANO seizure, and ASCO thrombosis guidance inform symptom and comorbidity management (69, 93, 94).	Regional reviews describe barriers within heterogeneous glioma populations. The availability of psycho-oncology, rehabilitation, and caregiver support is not consistently measured across countries (18, 19).

BTIP, Brain Tumor Imaging Protocol; GBM, Glioblastoma; PFS, progression-free survival; OS, overall survival.

## 4 Biological and molecular foundations

GBM is characterized by substantial genomic and cellular heterogeneity, with alterations affecting receptor tyrosine kinase/RAS/PI3K signaling, p53 regulation, and cell-cycle control. Common alterations include EGFR amplification or mutation, PTEN loss, CDKN2A/B deletion, TERT promoter mutation, PDGFRA amplification, and NF1 inactivation (6). These changes converge on overlapping growth and survival pathways, allowing tumor cells to adapt when individual pathways are inhibited.

Cellular states are also dynamic rather than fixed. GBM cells can transition between transcriptional states in response to genetic background, microenvironmental signals, and treatment pressure, contributing to intratumoral heterogeneity and therapeutic resistance (7, 8). This biology explains why activity against a single molecular target has rarely translated into durable benefit in unselected GBM populations.

Drug delivery presents an additional constraint. Contrast enhancement reflects blood–brain barrier disruption but does not imply uniform drug exposure throughout the tumor; infiltrative nonenhancing disease may remain relatively protected, while efflux transporters and regional vascular differences further influence tissue concentrations (24). Treatment itself can alter the remaining tumor, including the emergence of mismatch-repair defects and hypermutation after temozolomide exposure, although these changes do not reliably predict benefit from immune checkpoint inhibition (9).

## 5 Integrated diagnosis, biomarkers, and response assessment

### 5.1 Integrated diagnosis and molecular testing

Under WHO CNS5, GBM, IDH-wildtype, CNS WHO grade 4 is defined by microvascular proliferation or necrosis, or by EGFR amplification, TERT promoter mutation, or combined whole-chromosome +7/−10 in the appropriate clinicopathological setting (4, 5). In younger adults, midline tumors, or unusual histologies, IDH-mutant and H3-altered entities should be excluded before assigning a GBM diagnosis (5). Molecular classification is also essential for interpreting treatment evidence, as findings from IDH-mutant astrocytoma and high-risk low-grade glioma arise from distinct molecular entities and treatment settings and should not be directly extrapolated to GBM (25, 26).

MGMT promoter methylation remains the most clinically useful predictive biomarker for temozolomide benefit and is particularly relevant when treatment intensity is considered in older or frail patients (27–29). However, its predictive value in molecularly defined IDH-wildtype GBM remains uncertain. Results require assay-specific interpretation, and reports should identify the method, target region or CpG sites, cutoff, specimen adequacy, and any gray-zone result (5, 29). Methylation-based classification can resolve diagnostically difficult cases, while broader DNA/RNA sequencing is most useful when it clarifies an unusual diagnosis, identifies a relevant molecular alteration, or supports trial eligibility (30–32).

FGFR3–TACC3 fusions, BRAF V600E, and NTRK fusions may support diagnostic reassessment, trial referral, or consideration of tumor-agnostic therapy, but their interpretation depends on the assay and clinical context (32–36). Liquid biopsy has a more limited role: plasma cell-free DNA is often insensitive in primary brain tumors, whereas cerebrospinal-fluid ctDNA may better reflect intracranial tumor genomics in selected patients (37, 38).

In Mexico and Latin America, the immediate priority is reliable access to core molecular testing rather than universal broad sequencing. A small Mexican cohort reported a higher frequency of IDH2 alterations, highlighting a potential limitation of relying exclusively on IDH1-R132H immunohistochemistry, which does not detect IDH2 mutations or non-canonical IDH1 variants (39). When clinically indicated, molecular testing covering IDH1 and IDH2 should therefore be considered. Regional studies document uneven molecular characterization, supporting timely IDH and MGMT testing and defined pathways for additional assays when results may alter diagnosis, treatment, or trial eligibility (16, 17). The principal diagnostic and molecular requirements, together with practical considerations for their implementation in resource-variable settings, are summarized in **Table 2**.

**Table 2:** Diagnostic and imaging capabilities within the proposed care network.

Diagnostic and imaging capabilities within the proposed care network			
Function	Essential access	Enhanced specialist support	Comprehensive network capability
Tissue and reporting	Agree the tissue plan before sampling; preserve representative viable tissue for indicated assays; report adequacy and interpretation limits (5, 52).	Neuropathology review of limited, discordant, or low-confidence samples; documented specimen-transfer agreements.	Shared laboratory quality assessment, interoperable reports, and review of specimen failure or repeat sampling.
Integrated diagnosis	Histology and appropriate IDH assessment; MGMT by a validated assay; EGFR, TERT, or +7/−10 assessment when needed for classification (5, 27).	Methylation classification, copy-number interpretation, and specialist review for diagnostically difficult cases (30, 31).	Coordinated diagnostic pathways with transparent reporting and laboratory benchmarking; advanced tests used for a defined question.
Rare targets	Select testing according to morphology, age, clinical context, and a realistic diagnostic or treatment consequence (32–36).	Assay-aware molecular review; RNA-capable fusion testing; appraisal of CNS-specific versus tumor-agnostic evidence (32, 72, 73).	Shared eligibility review and outcome capture for rare-target treatment or trials; profiling is not automatically routine for every patient.
MRI acquisition	Comparable serial MRI with appropriate baseline and early postoperative imaging; retain source images and the corticosteroid record (3, 40, 42).	Expert second reads; perfusion, spectroscopy, or amino-acid PET for selected unresolved decisions (43).	Harmonized acquisition protocols, imaging quality assessment, and reliable transfer for trial or specialist review.
Response assessment	Interpret imaging with clinical status and steroid use; document uncertainty and use RANO 2.0 in the relevant context (42).	Adjudicate equivocal progression with multidisciplinary review and targeted additional imaging; iRANO is specific to immunotherapy contexts (43, 44).	Comparable longitudinal assessments and prospective adjudication records across participating centers.

IDH, isocitrate dehydrogenase; MGMT, O6-methylguanine-DNA methyltransferase; MRI, magnetic resonance imaging; CNS, central nervous system.

### 5.2 Imaging and response assessment

Pretreatment MRI should provide standardized T1, T2, FLAIR, diffusion, and post-contrast sequences, with perfusion when feasible (40). Early postoperative MRI establishes the surgical baseline; current SNO/EANO guidance recommends acquisition within 48 hours after surgery (3). RANO-Resect provides standardized reporting of residual enhancing and nonenhancing disease, while RANO 2.0 provides a framework for response assessment after treatment (41, 42). PET-RANO 1.0 can be applied when amino-acid PET is available, and iRANO remains relevant to immunotherapy studies (43, 44).

Advanced imaging should be used when it addresses a specific clinical question rather than as a routine substitute for standardized MRI. Perfusion, diffusion, spectroscopy, and amino-acid PET may help assess treatment effect or suspected progression, but their interpretation depends on acquisition, processing, timing, and clinical context (40, 42, 43). Consistent imaging protocols and access to expert review are therefore particularly valuable when findings are equivocal.

## 6 Surgery: maximal safe resection and functional preservation

The surgical objective is maximal safe cytoreduction while obtaining adequate tissue for integrated diagnosis and preserving neurological function. Greater resection of contrast-enhancing tumor is associated with improved outcomes, but the benefit must be weighed against postoperative deficits that may compromise independence and timely adjuvant treatment (41, 45). Extent of resection and postoperative neurological status should therefore be assessed together.

Functional mapping and intraoperative imaging address complementary aspects of surgical safety. Direct cortical and subcortical stimulation, including awake mapping when language or other functions require reliable intraoperative assessment, can help define functional boundaries; tractography and navigated transcranial magnetic stimulation may further inform planning when interpreted alongside the neurological examination and intraoperative findings (3, 46). Randomized trials support 5-aminolevulinic acid and intraoperative MRI for increasing complete resection of enhancing tumor (47, 48), while intraoperative ultrasound offers a more portable adjunct whose performance depends on image quality and operator experience (49). Sodium fluorescein may also be used in selected settings, although comparative evidence remains less mature (50).

Retrospective studies have associated resection beyond the contrast-enhancing margin with improved outcomes in selected patients, particularly with noneloquent disease, but definitions of supramaximal or supratotal resection vary and remain subject to anatomical and selection biases (45, 51). These data do not support a uniform target for removal of FLAIR abnormality. Reporting residual enhancing and nonenhancing disease together with postoperative neurological status provides a more meaningful assessment of the surgical result.

Biopsy remains appropriate when resection is unsafe because of patient condition, tumor location, or functional risk (3). Sampling should provide representative viable tissue for integrated diagnosis while minimizing procedural risk, with early communication with neuropathology when tissue is limited or an unusual diagnosis is suspected (5, 52). At recurrence, repeat sampling is most useful when it can clarify treatment effect, resolve diagnostic uncertainty, or inform a realistic treatment decision (32).

## 7 Radiotherapy across clinical scenarios

Postoperative radiotherapy with concurrent and adjuvant temozolomide remains the standard of care for adults with newly diagnosed GBM who are suitable for combined-modality treatment, typically delivered as 60 Gy in 30 fractions (1, 2). Treatment planning should incorporate the resection cavity, residual enhancing disease, operative findings, and relevant MRI abnormalities. ESTRO–EANO and ASTRO recommendations support anatomically defined target volumes, appropriate organ-at-risk constraints, image guidance, and systematic peer review (22, 23). IMRT and VMAT can improve conformity and organ-at-risk sparing, but treatment quality ultimately depends on accurate image registration, immobilization, and verification.

Age, performance status, comorbidity, and, particularly in older or frail patients, MGMT promoter methylation may inform the selection of treatment intensity. In older adults, 40 Gy in 15 fractions with temozolomide improved survival compared with the same radiotherapy alone, with greater benefit in MGMT-methylated disease (28). A separate randomized comparison of 25 Gy in 5 fractions and 40 Gy in 15 fractions supports the shorter regimen for selected elderly or frail patients within the studied population (53). Importantly, the randomized evidence supporting temozolomide in this setting derives from the 40 Gy in 15 fractions regimen; the 25 Gy in 5 fractions trial did not establish an equivalent benefit from adding temozolomide. These findings should not be extended to fit younger adults solely because travel is difficult. Patients who need a longer course require transport, accommodation, and continuity planning rather than an unsupported reduction in treatment.

Access to radiotherapy remains a practical constraint in Mexico. A national geospatial analysis estimated that approximately 29.5 million people live at least 100 km from a radiotherapy facility, while only about one third of the population lives in a municipality with a facility; most centers are concentrated in state capitals or Mexico City (15). These data describe geographic access rather than treatment delay or completion, but they highlight the burden of repeated treatment visits. For patients requiring referral, coordinated transport, accommodation, and remote treatment-planning review may help preserve timely delivery without unsupported treatment de-escalation.

Reirradiation is appropriate only for selected patients after review of the prior radiation plan, treatment interval, recurrence characteristics, functional status, and cumulative organ-at-risk exposure. ESTRO–EANO recommendations support individualized selection and stereotactic or hypofractionated approaches when appropriate (54). In RTOG 1205, reirradiation with concurrent bevacizumab improved progression-free survival compared with bevacizumab alone but did not improve overall survival (55). The expected clinical benefit and limitations of the evidence should therefore be considered before retreatment.

Dose intensification should be distinguished from improvements in treatment delivery. In INTRAGO-II, adding intraoperative radiotherapy to standard postoperative treatment did not improve progression-free survival, providing no basis for routine adoption of this strategy after resection (56).

For treatment planning, early postoperative MRI should be reviewed alongside subsequent imaging when cavity evolution or interval change affects target definition. Avoidable delays in starting radiotherapy should be investigated, particularly when they arise from postoperative recovery, planning, transport, or equipment constraints (3, 22, 23). **Table 3** summarizes the surgical and radiotherapy capabilities required for consistent delivery across different levels of service capacity.

**Table 3:** Surgical and radiotherapy capabilities and safety responsibilities.

Surgical and radiotherapy capabilities and safety responsibilities			
Function	Essential access	Enhanced specialist support	Comprehensive network capability
Operative selection	Choose resection or biopsy according to clinical condition, functional risk, diagnostic need, and care goals (3).	Refer when mapping, a deep or eloquent approach, or reoperation requires expertise unavailable locally (3, 46).	Shared case review and risk-adjusted assessment of postoperative function, tissue yield, and access to adjuvant treatment.
Intraoperative assessment	A function-preserving strategy and adequate diagnostic sampling; ultrasound may be a practical adjunct where expertise exists (49, 52).	Direct stimulation, fluorescence, or intraoperative MRI selected for the operative question and patient (46–48, 51).	Coordinated specialist access and technical review. Greater resection or greater technology use is not itself a quality target.
Postoperative assessment	Document neurological status and obtain early MRI; assess residual enhancing and nonenhancing disease (3, 41).	Volumetric RANO-Resect reporting and review when the interpretation is uncertain (41).	Shared imaging-quality checks and longitudinal functional-outcome records.
RT planning and delivery	Indicated dose/fractionation, CT–MRI registration, anatomically edited targets, OAR constraints, verification, and TMZ coordination (1, 22, 23).	IMRT/VMAT planning support, appropriate IGRT, and pretreatment peer review; specialist escalation for complex plans.	Harmonized technical QA, plan archives, and outcome review; replanning responds to a clinical or dosimetric need, not a technology mandate.
Older or frail adults	Select treatment by fitness, MGMT, goals, and the relevant trial evidence; avoid using travel burden alone to justify de-escalation (3, 28, 53).	Functional assessment and coordinated transport, lodging, toxicity review, and caregiver support.	Evaluate treatment completion, function, hospitalization, and burden across the pathways actually delivered.
Reirradiation QA	Retrieve prior RT records; document the indication, goals, and multidisciplinary selection (54, 55).	Assess prior and proposed dose distributions, cumulative OAR exposure, target geometry, and treatment-specific QA (54).	Central dosimetry review and prospective toxicity/outcome capture. Clinical selection is addressed in the recurrence pathway.

RT, radiotherapy; TMZ, temozolomide; OAR, organ at risk; QA, quality assurance; IGRT, image-guided radiotherapy; IMRT/VMAT, intensity-modulated/volumetric-modulated arc therapy.

## 8 Systemic and device-based therapy for newly diagnosed disease

Concurrent temozolomide followed by six adjuvant cycles remains the systemic backbone of postoperative chemoradiotherapy (1, 2). MGMT promoter methylation helps estimate expected benefit, alongside functional status, comorbidity, hematological tolerance, and patient preference (27, 29).

In CATNON, a *post-hoc* molecular analysis identified a subgroup meeting current criteria for IDH-wildtype GBM in which adding temozolomide to radiotherapy did not demonstrate an overall or progression-free survival benefit; MGMT promoter methylation was prognostic but did not show a predictive interaction with temozolomide benefit in this subgroup (57). The 2026 final CATNON analysis confirmed a benefit from adjuvant temozolomide in IDH-mutant astrocytoma (median overall survival, 12.5 vs. 6.0 years; HR 0.54, 95% CI 0.42–0.69), but not from concurrent temozolomide (9.7 vs. 7.2 years; HR 0.81, 95% CI 0.63–1.04); no benefit from either schedule was observed in IDH-wildtype tumors (25). In the molecular analysis of NRG Oncology/RTOG 9802, 26 of 106 patients with available tissue had IDH-wildtype tumors, with no significant benefit from adding procarbazine, lomustine, and vincristine to radiotherapy; however, these patients were enrolled in a high-risk low-grade glioma trial, not a GBM trial (26). These findings should not be extrapolated across molecular entities, tumor grades, or chemotherapy regimens, and do not independently establish a change in the current treatment approach for GBM.

Beyond the choice of regimen, optimizing temozolomide exposure is also important. Dose-dense schedules increased toxicity without improving survival, while current evidence does not support routinely extending adjuvant treatment beyond six cycles (58).

Lomustine-temozolomide produced longer overall survival than temozolomide alone in the selected MGMT-methylated population of CeTeG/NOA-09, but its small, open-label design and hematological toxicity limit its use as a general alternative to standard temozolomide (59). Bevacizumab added to initial chemoradiotherapy improved progression-free but not overall survival and is therefore not routinely recommended in newly diagnosed disease (60, 61).

Tumor-treating fields (TTFields) added to maintenance temozolomide improved progression-free and overall survival in EF-14 (62). Greater device use was associated with longer survival in *post hoc* analyses, although this does not establish a randomized benefit of a particular adherence threshold (63). Its use also depends on scalp toxicity, patient and caregiver support, logistics, and affordability, making treatment acceptance and actual use relevant to assessing delivery.

In older or medically complex patients, treatment selection should incorporate functional status, MGMT methylation, comorbidity, cognition, expected treatment burden, and patient goals rather than age alone (3, 28). Treatment delivery should distinguish prescribed therapy from completed therapy. Recording dose delays, reductions, completed cycles, hematological recovery, and reasons for discontinuation can identify gaps between the intended regimen and the treatment actually received.

## 9 Management at recurrence

At recurrence, treatment decisions should begin with confirmation of progression, since pseudoprogression, radiation injury, postoperative change, seizure-related findings, and corticosteroid-associated imaging effects can mimic disease progression (42). Once recurrence is established, management depends on functional status, symptoms, disease distribution, previous treatment, molecular context, and patient goals.

Reoperation may provide cytoreduction, symptom relief, or new tissue in selected patients with accessible focal recurrence, while reirradiation may be considered after review of prior dosimetry, treatment interval, target characteristics, and cumulative organ-at-risk exposure (3, 54, 55). Both approaches are selection dependent and should be weighed against expected functional benefit and treatment burden.

Lomustine remains a commonly used salvage option and trial comparator (3, 64). Bevacizumab can reduce edema, corticosteroid requirements, and radiographic enhancement, but randomized studies have not established an overall-survival benefit (64, 65). Temozolomide rechallenge may be considered selectively, particularly in patients with a treatment-free interval and MGMT-methylated tumors, although evidence remains limited (3).

REGOMA reported longer survival with regorafenib than lomustine in phase 2 (66), whereas the larger GBM AGILE platform did not confirm superiority and identified additional toxicity (67). Regorafenib should therefore not be presented as a routine preferred standard. Similarly, TTFields did not improve overall survival over physician-choice chemotherapy in recurrent disease and should not be extrapolated from EF-14 as an established salvage standard (68).

Clinical-trial referral should be considered while functional status remains adequate, particularly when eligibility depends on molecular findings, corticosteroid exposure, specialized procedures, or treatment logistics (3). Supportive and palliative care should remain integrated throughout recurrence management rather than reserved for patients who no longer receive active treatment (69). Current recurrence decisions and the supportive-care and referral capabilities required to deliver them are summarized in **Table 4**.

**Table 4:** Recurrence decisions and supportive-care responsibilities across capability levels.

Recurrence decisions and supportive-care responsibilities across capability levels			
Decision or need	Essential assessment and access	Enhanced specialist support	Comprehensive network capability
Confirm progression	Review MRI, neurological status, steroid use, and possible treatment-related or seizure-related changes before changing treatment (42).	Expert neuroradiology review and advanced imaging when an unresolved question can change management (43).	Shared adjudication processes and prospective recording of uncertainty and its resolution.
Fitness and patient goals	Record function, symptoms, cognition, steroid requirement, comorbidities, and goals; consider caregiver capacity (3, 69).	Neurorehabilitation, psycho-oncology, complex symptom management, and navigation when required.	Longitudinal patient- and caregiver-reported outcomes linked to the clinical pathway.
Local treatment	Consider reoperation or reirradiation in selected patients with a defined clinical objective; discuss expected benefit and uncertainty (3, 54, 55).	Surgical or reirradiation board review with current imaging and prior treatment records.	Comparable selection and outcome records; specialist access without unnecessary transfer of all subsequent care.
Systemic and molecular options	Consider salvage options in context. Bevacizumab is often symptom-directed; REGOMA was not confirmed by GBM AGILE, so regorafenib is not a routine preferred standard (64–67).	Repeat tissue or molecular testing only when it can resolve diagnosis or enable a feasible treatment/trial pathway (32).	Shared rare-target review, pharmacovigilance, and prospective documentation of treatment rationale and outcomes.
Trial access	Consider trials before avoidable loss of eligibility; assess function and relevant molecular requirements (3).	Dedicated review of steroid limits, travel, consent, manufacturing, coverage, and caregiver feasibility.	A trial network using shared pathology, imaging, RT QA, and transparent screening/nonenrollment denominators.
Supportive and palliative care	Provide symptom, seizure, metabolic, mental-health, rehabilitation, and caregiver assessment throughout the disease course (3, 69, 93).	Specialist support for complex symptoms, anticoagulation, psychiatric risk, and advance-care planning (69, 94, 109).	Coordinate hospital and community care with patient-valued outcomes. Palliative care is available at every capability level.

MRI, magnetic resonance imaging; RT, radiotherapy; QA, quality assurance.

## 10 Emerging therapeutic strategies

### 10.1 Targeted therapy and the limits of single-pathway selection

EGFR amplification and EGFRvIII have been extensively investigated, but phase 3 trials of depatuxizumab mafodotin and rindopepimut failed to improve survival (70, 71). Heterogeneous target expression, antigen loss, redundant signaling, and limited drug delivery may contribute to these results (6–9, 24). Molecular selection should therefore be based not only on the presence of an alteration, but also on evidence that it predicts treatment response in the relevant disease setting.

Dabrafenib plus trametinib has shown activity in BRAF V600E-mutant high-grade glioma, while larotrectinib has demonstrated activity in TRK-fusion-positive CNS tumors (72, 73). These data come from small or basket populations and should not be extrapolated to unselected adult GBM. FGFR3–TACC3 fusions and selected MET alterations may support trial referral or molecularly guided treatment in appropriate settings, but their detection alone does not establish a routine GBM indication (32–36).

### 10.2 Immune checkpoint inhibition, vaccines, and oncolytic virotherapy

Checkpoint inhibition has not established a role in routine GBM care. Nivolumab failed to improve survival over bevacizumab in recurrent disease and did not improve outcomes when added to standard treatment in newly diagnosed tumors, whether MGMT was unmethylated or methylated (74–76). Dual ipilimumab-nivolumab also failed to improve progression-free survival over temozolomide in newly diagnosed MGMT-unmethylated disease (77). A phase 1 study of anti-LAG-3 therapy with or without nivolumab demonstrated feasibility and exploratory immune activity, but was not designed to establish efficacy (78).

Personalized peptide and DNA vaccines have generated neoantigen-specific immune responses in early-phase studies, but clinical benefit remains uncertain (79–81). DCVax-L reported longer survival than an external control, although interpretation is limited by the study design and control-arm methodology (82). Oncolytic approaches have similarly produced durable responses in selected patients, including studies of poliovirus and DNX-2401-based therapies; teserpaturev has received conditional approval in Japan under a specific regulatory pathway, but these findings do not establish a generalizable standard for Latin America (83–85).

### 10.3 Cellular therapy, DNA-damage response, and blood–brain-barrier modulation

Locoregional CAR-T approaches targeting IL13Rα2 or EGFR/IL13Rα2 have shown intracranial activity in early-phase studies (86, 87). Their clinical development remains limited by antigen heterogeneity, persistence, neurotoxicity, manufacturing requirements, and the need for specialized intracranial delivery and monitoring. These approaches should therefore remain within appropriately designed clinical trials.

DNA-damage-response strategies have likewise not established a new standard. Veliparib failed to improve outcomes in MGMT-unmethylated GBM, while olaparib combinations have provided mainly pharmacokinetic and safety data, including relevant hematological toxicity (88, 89). ATR inhibition remains largely preclinical in the evidence cited here (90). Ultrasound-mediated blood–brain-barrier opening has been evaluated with albumin-bound paclitaxel and carboplatin in early-phase recurrent GBM studies, demonstrating feasibility and altered drug delivery without an established survival benefit (91, 92).

The appropriate regional response to these technologies is selective trial access rather than premature routine adoption. Their evaluation requires appropriate molecular and imaging workflows, specialized procedures where applicable, and prospective assessment of efficacy, safety, and patient-valued outcomes. **Figure 2** summarizes the relationship between evidence maturity and the delivery requirements of established, specialist-supported, and investigational approaches.

**Figure 2:**
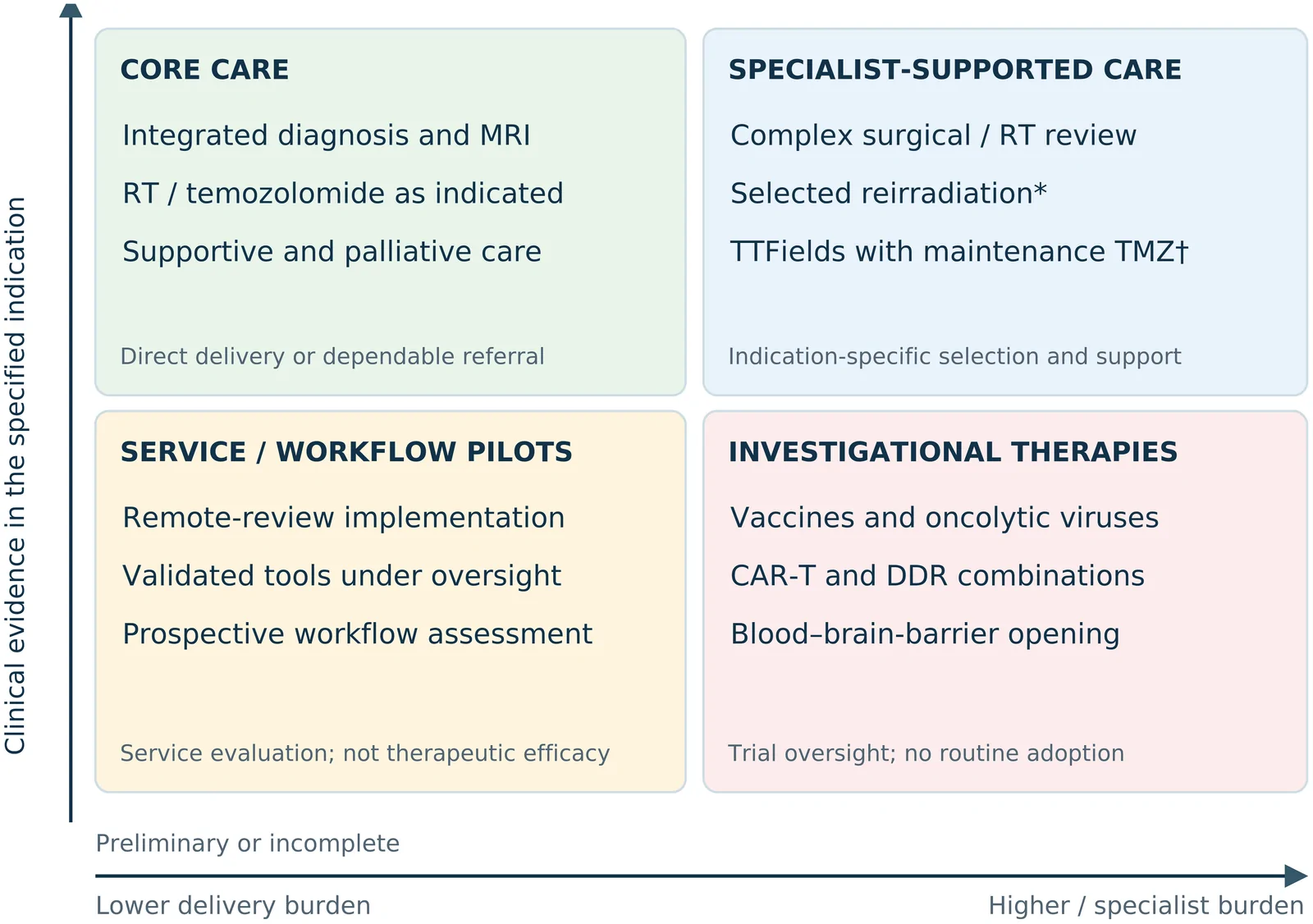
Evidence maturity and delivery requirements for glioblastoma care. Interventions are grouped according to the maturity of supporting evidence and the expertise, infrastructure, and oversight required for their delivery. Established care may be provided locally or through reliable referral, whereas selected specialist interventions require individualized assessment and investigational approaches require appropriate clinical-trial oversight. The categories are qualitative and indication-specific and do not represent rankings of efficacy, cost, or geographic feasibility. *Selected recurrence: PFS benefit in RTOG 1205; no OS benefit established. † Newly diagnosed disease after chemoradiation; support and affordability matter. PFS, progression-free survival; OS, overall survival; DDR, DNA-damage response.

## 11 Supportive care, comorbidities, and palliative care

Supportive care is integral to GBM management from diagnosis onward, with the goals of preserving neurological function, maintaining treatment continuity, controlling symptoms, and protecting quality of life. Dexamethasone can provide rapid relief of symptomatic edema but should be used at the lowest effective dose and tapered when clinically feasible because toxicity increases with dose and duration (3, 69). Routine antiseizure prophylaxis is not recommended in patients who have never had a seizure; when treatment is indicated, non–enzyme-inducing agents are generally preferred because of their lower potential for drug interactions (93).

Quality of life is influenced not only by tumor burden but also by fatigue, cognitive and psychiatric symptoms, language or motor impairment, sleep disturbance, caregiver burden, and treatment toxicity. These concerns should be assessed alongside performance status and radiographic response, with rehabilitation, psycho-oncology, neuropsychology, social work, and caregiver support incorporated according to individual needs (69). Venous thromboembolism requires individualized assessment of thrombosis and intracranial bleeding risk, particularly after neurosurgery or in the presence of thrombocytopenia (94). Comorbidities such as diabetes, hypertension, renal dysfunction, and impaired wound healing should likewise be considered when treatment or corticosteroid exposure is modified.

Early palliative care can address symptom control, communication, advance-care planning, and caregiver needs while disease-directed treatment continues (69). In EPCOG, the prespecified six-month quality-of-life endpoint was not met; although some secondary symptom and mood measures favored the intervention, overall survival was shorter in the intervention group (95). These findings should not be interpreted as evidence against palliative care itself, but they do argue against assuming that earlier specialist involvement necessarily improves survival or quality of life without considering the specific intervention and its context.

Supportive and palliative care should therefore remain integrated throughout the disease course, including during active treatment and at recurrence. Treatment decisions should account for function, symptoms, cognitive status, caregiver capacity, and patient priorities rather than relying on survival or imaging outcomes alone.

## 12 Artificial intelligence and radiomics in clinical practice

Artificial intelligence (AI) and radiomics are increasingly being evaluated for GBM imaging, but predictive performance alone does not establish clinical utility. Potential applications include segmentation, volumetric assessment, longitudinal response measurement, and differentiation of pseudoprogression from progression. Their value depends on whether they improve a defined clinical decision or workflow rather than on model accuracy alone (96–100).

Radiomic features should be standardized according to the Image Biomarker Standardisation Initiative, while prediction models should follow appropriate reporting and appraisal standards such as TRIPOD+AI, PROBAST+AI, and CLAIM (96–99). These frameworks improve transparency and assessment of methodological quality but do not establish clinical benefit. The available GBM study on pseudoprogression illustrates this distinction: its retrospective design and reported use of synthetic oversampling before the training/test split raise concerns about information leakage and limit its readiness for clinical use (98, 100).

Clinical implementation should therefore require independent validation, assessment across relevant scanners and patient groups, comparison with existing clinical practice, and prospective evaluation of its effect on management. In Mexico, translation into practice may be further constrained by the limited availability of multidisciplinary teams integrating clinical specialists with quantitative scientists, as well as the lack of well-structured, comprehensive datasets combining clinical, imaging, pathological, radiotherapy, and chemotherapy information from Mexican patients. Collaborative models such as the Integrated Mathematical Oncology group at Moffitt Cancer Center illustrate the value of this type of interdisciplinary approach (101). Developing such teams and datasets would support the development, validation, and clinical translation of AI and radiomics tools for Mexican patients with GBM. AI should complement rather than replace tissue diagnosis, expert imaging interpretation, multidisciplinary review, or clinician responsibility. **Figure 3** summarizes a human-supervised evaluation pathway from model development and validation to clinical assessment and post-deployment monitoring.

**Figure 3:**
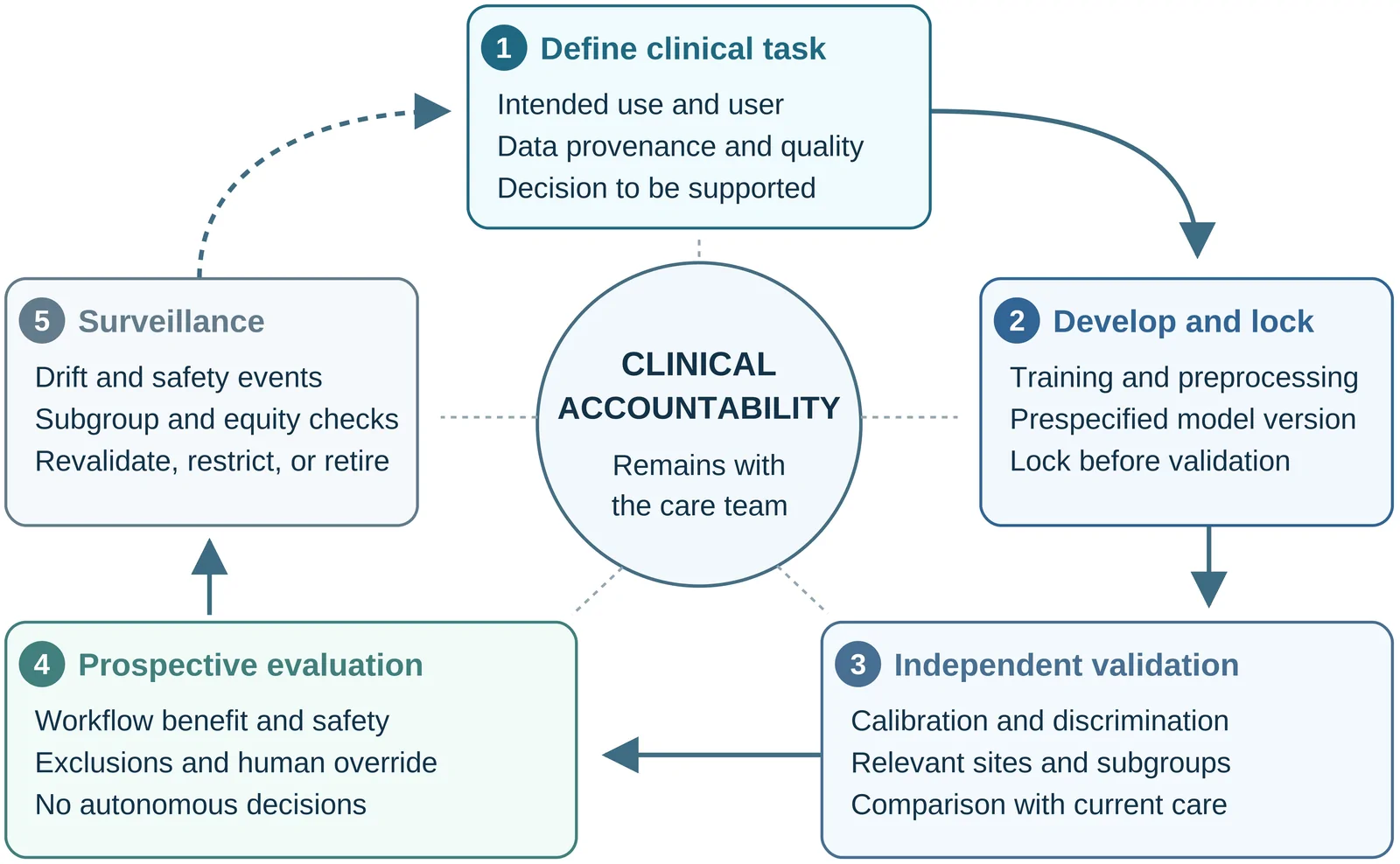
Human-supervised lifecycle for clinical AI and radiomics in glioblastoma care. The framework links definition of the clinical task and intended use with model development, independent validation, prospective workflow evaluation, supervised deployment, and post-deployment surveillance. Clinician oversight, accountability, and predefined criteria for modification, restriction, or withdrawal are maintained throughout the lifecycle. IBSI, TRIPOD+AI, PROBAST+AI, and CLAIM inform complementary aspects of imaging standardization, reporting, and model appraisal.

## 13 Evidence-to-implementation roadmap for Mexico and Latin America

A regional care network should begin by defining how patients obtain each essential component of care, from tissue diagnosis and molecular testing to standardized imaging, appropriate treatment, and supportive care. Some functions can be provided locally, whereas others require dependable referral to a specialist center. Referral agreements should define who sends the material or patient, who receives it, the expected response time, and who is responsible for acting on the result.

Regional evidence can help identify where these arrangements are most needed, but its limits should remain explicit. Brazilian multicenter data show differences in molecular testing between public and private care, while an Argentine tertiary-center cohort illustrates incomplete molecular classification as diagnostic requirements increase (16, 17). Mexico contributes national data on radiotherapy access and clinical experience, whereas Chile contributes single-center outcome data (12–15). These studies inform specific barriers but cannot establish service availability or expected outcomes across countries that were not represented.

The referral model presented in **Figure 4** is bidirectional by design. Local teams can retain routine treatment when quality requirements are met, while referral hubs provide complex surgery, advanced neuropathology, difficult treatment planning, recurrence review, and access to specialized trials. Imaging, specimens, and treatment plans can often be reviewed remotely, avoiding unnecessary transfer of the entire course of care. When travel is required, the pathway should include appropriate logistical support and a defined plan for return to local care.

**Figure 4:**
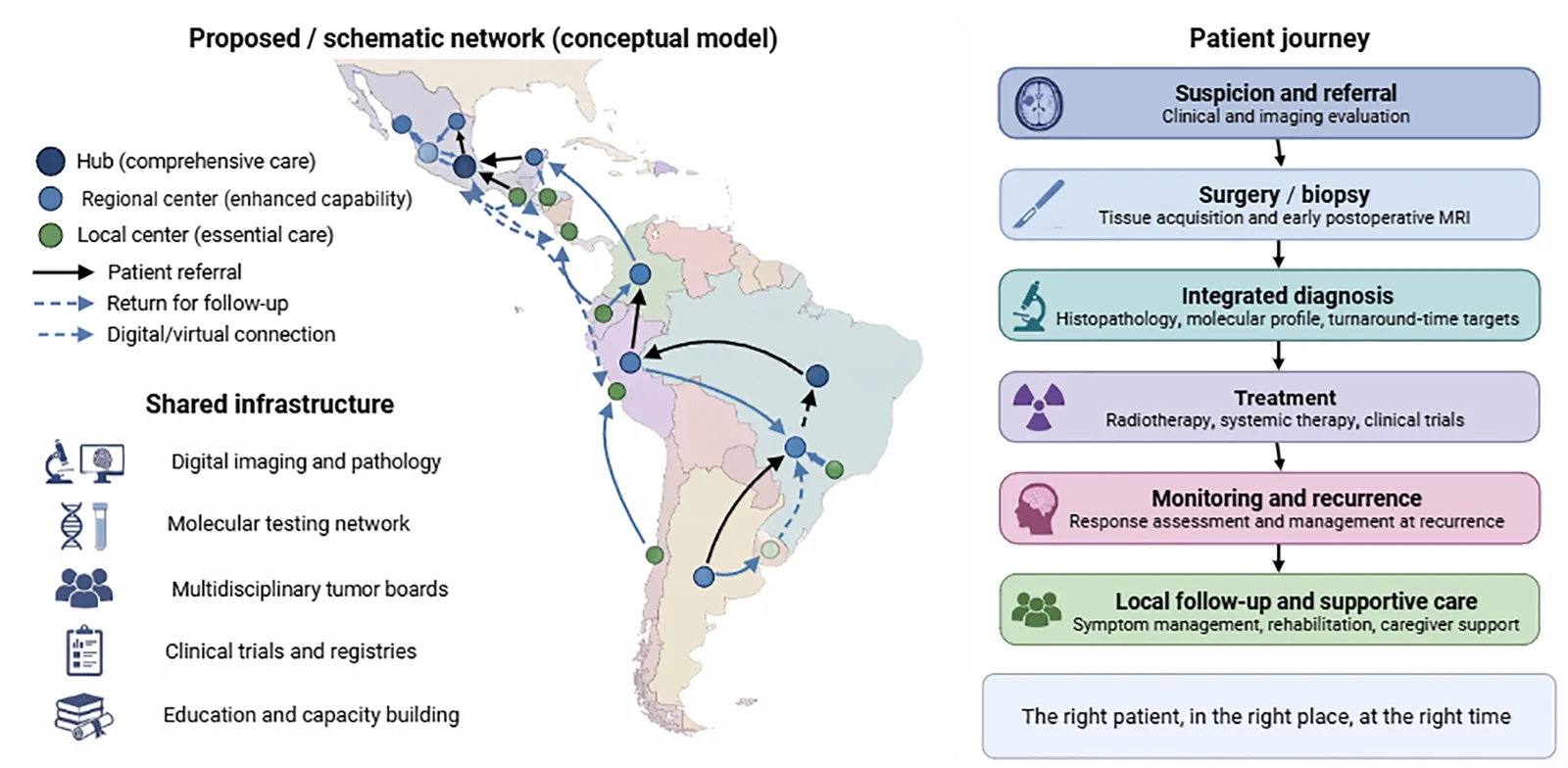
Proposed regional care network for glioblastoma. The model combines local care with selective referral to specialist hubs when the required expertise or infrastructure is not available locally. Imaging, specimens, and treatment plans may be reviewed remotely, whereas procedures requiring in-person expertise are performed at referral centers, followed by return to the closest qualified service. The pathway is schematic and does not represent verified institutions, geographic coverage, or existing referral relationships. Postoperative imaging and laboratory processes may proceed in parallel, and urgent care should not await completion of every step. Supportive and palliative care remain available throughout. MRI, magnetic resonance imaging. Created in BioRender. Rodriguez Camacho, A (2026).. https://BioRender.com/e4gpue.d .

Implementation should be evaluated as a set of defined interventions rather than as an undifferentiated network package. Frameworks such as CFIR and RE-AIM can help characterize contextual barriers, reach, adoption, implementation, and maintenance (102, 103), while each intervention should specify the process being changed, the responsible team, the setting, and the support required (104). Prospective registries, hybrid effectiveness-implementation studies, and pragmatic or stepped-wedge evaluations could test selected components under appropriate conditions. **Table 5** provides operational definitions and candidate targets for prospective evaluation. These targets are author-proposed implementation measures and should not be interpreted as validated clinical thresholds.

**Table 5:** Operational definitions and candidate targets for prospective network evaluation.

Operational definitions and candidate targets for prospective network evaluation			
Indicator	Start/end event or denominator	Candidate target	Basis and safeguards
Integrated-report TAT	Calendar days from surgery/biopsy to a signed integrated report; retain incomplete, failed, or pending cases and record transfer stages.	Illustrative local SLA: ≤14 days for local testing; ≤21 days for send-out.	Author-proposed service-planning examples intended to return results before relevant decisions. No validated survival cutoff. Report median/P90, completeness, and time at each stage.
Core molecular completeness	Patients with all clinically indicated diagnostic and treatment-informing results available for the first definitive postoperative decision/eligible patients.	No universal percentage proposed.	Describe missing tests and consequences; diagnostic reflex requirements are not optional because a performance percentage has been reached.
External-test performance	Tests returned within the explicitly agreed laboratory SLA/all tests sent.	Locally negotiated SLA; no universal ≥90% target.	Report failures, repeat tests, tissue exhaustion, and elapsed time without resetting the clock. Calibrate against local baseline and clinical need.
Early postoperative MRI	Resected patients with MRI by 48 hours/resected patients eligible for postoperative MRI.	≤48 hours; proposed ≥90% completion.	The clinical interval follows SNO/EANO guidance (3); the completion percentage is author-selected. Report justified exceptions and later scans separately.
Persistent neurological deficit	New or worsened deficits still present 30 days after surgery/operated patients with follow-up.	Risk-adjusted monitoring; no fixed ceiling.	Interpret function with residual tumor and subsequent treatment eligibility. Thirty days is the audit assessment point, not a treatment threshold.
RT initiation	Calendar days: surgery to first fraction; report all patients intended for RT, including those never started.	Proposed ≥80% by 6 weeks.	An operational target, not a proven causal cutoff. Record recovery, clinical readiness, progression, referral, and planning delays (3, 22, 23).
Pretreatment peer review	Plans reviewed before the first fraction/eligible plans.	Proposed ≥90%.	Author-proposed. Record the reason for omissions and whether review changed the plan; define urgent-treatment exceptions.
Avoidable RT interruption	Courses with a preventable unplanned break/courses started; record duration and attributed cause.	No universal ≤5% ceiling.	Classify equipment, travel, access, toxicity, and other causes. Report completion and abandonment separately; do not label all medically necessary breaks preventable.
Recurrence decision time	Business days from referral for suspected recurrence to a documented multidisciplinary recommendation.	Optional planning example: ≤10 business days.	Author-proposed scheduling example, not an efficacy threshold. Additional imaging/biopsy and urgency are recorded; deterioration bypasses routine scheduling.
Decision-to-treatment time	Calendar days from recurrence recommendation to the chosen active treatment; retain all decisions.	Optional planning example: ≤14 calendar days.	Author-proposed access example. Observation, further investigation, and symptom-focused care are separately defined pathways, not automatic failures.
Supportive-care assessment	Applicable steroid, seizure, glucose, distress, rehabilitation, caregiver, and palliative needs assessed.	No universal ≥80% threshold.	Author-proposed process target. Report components and response to identified need. A completed checklist without accessible care is insufficient.
Equity and data completeness	Required registry fields completed; referral completion, support use, and abandonment stratified by site/payer/travel.	Report missingness and within-network differences; no universal ≥90% threshold.	Author-proposed. Retain untreated patients, missing data, and catchment definitions. Expansion should not improve completeness by excluding harder-to-follow patients.

Evidence- or guideline-based intervals are distinguished from author-proposed candidate targets. Author-proposed numerical thresholds are intended as operational indicators for local assessment and prospective validation and should not be interpreted as established performance standards. TAT, turnaround time; P90, 90th percentile; SLA, service-level agreement; RT, radiotherapy; MRI, magnetic resonance imaging.

The minimum regional dataset should remain small enough to complete. Suggested fields include age, sex, performance status, symptom and diagnostic dates, surgery or biopsy date, RANO-Resect class, IDH and MGMT results, radiotherapy dose and dates, treating center, payer, travel distance or time, temporary relocation, transportation or lodging support, temozolomide exposure, TTFields use and adherence when applicable, recurrence date and confirmation method, steroid dose, seizures, venous thromboembolism, major comorbidities, palliative-care referral, trial screening, treatment interruption or abandonment, and survival. Equity metrics should be stratified by institution, payer, geography, and socioeconomic proxy.

Research access should also be measured using transparent denominators, including patients diagnosed, screened, eligible, approached, enrolled, treated, and followed, with screen failures and nonenrollment recorded. This allows research activity to be interpreted in relation to the population eligible for participation rather than simply the number of patients enrolled at referral centers.

## 14 Discussion

The central inference from this review is that realized prognosis in GBM reflects both tumor biology and the reliability of the care pathway. A GBM-specific meta-analysis of 24 studies including 2,552 patients from low- and middle-income countries reported a pooled median overall survival of 14.17 months, increasing from 12.54 months in studies predating widespread adoption of the Stupp regimen to 15.64 months in later studies, without a significant difference according to World Bank income category (20). In contrast, a separate meta-analysis of 12 studies including 143,303 patients found that lower individual economic status was associated with poorer survival (pooled HR 1.09, 95% CI 1.02–1.17) (21). These findings are not contradictory: national income is a coarse ecological measure, whereas individual socioeconomic disadvantage and failure to complete successive diagnostic and treatment steps are more proximal to the care actually received. The “survival gates” described in low- and middle-income settings therefore provide plausible mechanisms through which structural disadvantage may translate into poorer outcomes, but they should not be interpreted as a causal decomposition of survival (18, 19).

Contemporary data from other settings provide useful external benchmarks, although differences in population selection and study design limit direct comparisons. A regional Spanish study of 530 patients with IDH-wildtype GBM diagnosed between 2011 and 2021 reported a median overall survival of 12.3 months (105). Similarly, a recent real-world cohort of 832 adults with WHO 2021-defined IDH-wildtype GBM treated with radiotherapy, with or without chemotherapy, reported a median overall survival of 13.0 months; its analysis also distinguished patients meeting trial-eligibility criteria from the broader treated population (106). The 2018–2022 CBTRUS report provides a complementary population-based benchmark, with a five-year relative survival of 7.0% among patients of all ages diagnosed with GBM in 2004–2021. Although 82.1% of GBM cases in the report’s molecular-marker analysis were coded as IDH-wildtype, the survival estimate derives from a historical cohort without uniform molecular characterization, limiting direct comparison with contemporary WHO CNS5-defined cohorts (107). Taken together, these studies illustrate the importance of distinguishing molecularly defined regional cohorts, real-world treatment outcomes, and population-level epidemiological estimates rather than treating them as directly interchangeable.

Regional molecular data illustrate how these gaps can become clinically consequential. LACOG 0619 included 828 adults from nine Brazilian centers; GBM represented 57% of the cohort, and fewer than 20% of eligible patients underwent IDH sequencing, with marked differences in access to IDH, 1p/19q, MGMT, and next-generation sequencing between public and private institutions (16). An Argentine cohort of 443 surgically treated patients likewise found incomplete molecular characterization under WHO CNS5, reaching 36% of assessed cases in 2024 (17). These cohorts include broader glioma populations and cannot be extrapolated directly to all adult GBM, but they expose that diagnostic standards can become more precise while their application remains incomplete. The clinical value of a reference laboratory therefore depends not simply on assay availability, but on adequate tissue, dependable transfer, interpretable reporting, appropriate turnaround, and communication with the team making the treatment decision.

The same principle applies to centralization. Mexican geospatial data demonstrate substantial geographic separation from radiotherapy services, but distance alone does not measure waiting time, treatment capacity, payer eligibility, machine availability, or treatment completion (15). Concentrating expertise may improve molecular interpretation, complex surgery, radiotherapy planning, or recurrence review, while requiring every treatment visit at a distant hub may increase financial and logistical burden. The relevant objective is therefore not maximal centralization, but reliable access to the expertise required for each decision. Local treatment with specialist review may be preferable to complete transfer when quality requirements can be met. A network should be judged by whether it preserves the benefit of specialist care without creating new barriers to completing treatment.

The therapeutic history of GBM shows that biological plausibility, molecular enrichment, and early activity do not reliably predict population-level benefit. Negative phase 3 studies of EGFR-directed approaches and immune checkpoint inhibition illustrate the limits of extrapolating from mechanism or preliminary activity to routine care (70, 71, 74–77). Economic studies further demonstrate substantial variation in GBM costs across health systems, although differences in methods and included services limit direct comparison (108). Adoption decisions should therefore consider not only whether a technology can be obtained, but the magnitude and certainty of its expected benefit, the patients who are likely to benefit, the infrastructure and workforce required, and the opportunity cost to existing services.

The proposed roadmap is consequently intended as a delivery and evaluation framework rather than an alternative treatment guideline. Its central test is whether essential clinical functions can be completed more reliably while preserving patient autonomy and minimizing avoidable travel, delay, duplication, and treatment abandonment. The operational targets in **Table 5** should be treated as candidate benchmarks rather than validated thresholds. Their value depends on what they measure: faster reporting is useful only if the report is complete and reaches the responsible team; faster referral is useful only if it does not increase duplicate testing or loss to follow-up. Evaluation should therefore include balancing measures and the reasons for exceptions rather than rewarding an isolated percentage.

The regional evidence base itself remains an important limitation to interpretation. Published data are concentrated in a small number of countries and frequently originate from tertiary centers, leaving rural populations, different payer groups, and patients lost before pathological confirmation underrepresented. A regional registry could improve benchmarking only if it records its catchment population, uses compatible definitions, and retains patients whose care is interrupted. Importantly, research access should be evaluated using the same logic: the number enrolled in trials is insufficient without knowing how many patients were diagnosed, screened, eligible, approached, treated, and followed. Otherwise, referral-center activity may appear successful while the population with potentially limited access remains invisible.

Initial implementation studies should therefore test defined components of the network rather than attribute changes in survival to the framework as a whole. Diagnostic completeness, treatment completion, functional outcomes, patient burden, and equity can be evaluated together with adjustment for clinical risk and secular changes. A useful finding may be that a particular service should remain local, be centralized, or be redesigned. This approach provides a stronger basis for expansion than assuming that greater technological intensity, referral volume, or institutional complexity necessarily represents better care.

## 15 Limitations

This review has several limitations. The literature selection was narrative and may be affected by citation and selection bias. The evidence base also varies in diagnostic classification, age, treatment era, and health-system setting; several regional studies include mixed glioma populations or historical GBM cohorts and therefore cannot be assumed to represent adult IDH-wildtype GBM as currently defined.

The regional evidence is geographically and institutionally uneven, with limited population-based molecularly annotated datasets outside a small number of countries and substantial representation from tertiary centers. Consequently, the magnitude of unmet need, treatment abandonment, and within-country inequity cannot be estimated reliably across Latin America. The absence of data from a country should not be interpreted as evidence of equivalent access or outcomes.

The referral model, capability levels, and operational targets proposed here have not been prospectively validated as a package. Regulatory status and reimbursement were not audited country by country, and no formal cost-effectiveness analysis was performed. Implementation will require local assessment of governance, financing, geography, workforce, and existing referral capacity. The tables and figures are intended to support such evaluation; they are not a formally graded clinical guideline.

## 16 Conclusion

Improving outcomes for adults with glioblastoma in Mexico and Latin America will depend not only on advancing therapies, but on making the benefits of existing evidence-based care consistently attainable. The central priority is therefore to build systems in which molecular diagnosis, specialized expertise, treatment, supportive care, and research access remain connected despite geographic, socioeconomic, and institutional differences. Rather than defining equity by the technologies a center can provide, a more meaningful standard is whether every patient can enter an appropriate pathway, reach the level of expertise their disease requires, and complete care without avoidable barriers. This approach reframes regional capacity not as a limitation to be accepted, but as a system that can be deliberately measured, strengthened, and evaluated.
