# Supplementary material for: Adult IDH-wildtype glioblastoma in Mexico and Latin America: contemporary standards, emerging strategies, and an evidence-to-implementation roadmap
Source: Front Oncol. 2026 Oct 28;16:1998982. doi: 10.3389/fonc.2026.1998982 (PMC13752682; doi:10.3389/fonc.2026.1998982)
Supplement: Supplementary file 1 [file Table1.docx]

**Supplementary Material**

**Literature Search Strategy**

**Search overview**

**Search period:** January 1, 2005, to August 19, 2026.

**Languages:** English and Spanish.

**Databases and sources:** PubMed/MEDLINE, Scopus, Web of Science, GLOBOCAN, major society guidelines, consensus statements, and regional policy and health-system capacity reports.

**Review type:** Structured narrative review.

**1. Main search concepts**

Search combined disease-specific terms with molecular, clinical, treatment, implementation, and geographic concepts as appropriate to each database.

**2. Search strategy**

**2.1 PubMed/MEDLINE**

("Glioblastoma"[Mesh] OR "glioblastoma"[Title/Abstract] OR "glioblastoma multiforme"[Title/Abstract]) AND ("IDH-wildtype"[Title/Abstract] OR "IDH wild-type"[Title/Abstract] OR "IDH1 wild-type"[Title/Abstract] OR "IDH1-wildtype"[Title/Abstract] OR "WHO CNS5"[Title/Abstract]) AND ("molecular diagnosis"[Title/Abstract] OR "molecular classification"[Title/Abstract] OR "integrated diagnosis"[Title/Abstract] OR "MGMT"[Title/Abstract] OR "EGFR"[Title/Abstract] OR "TERT"[Title/Abstract] OR "chromosome 7"[Title/Abstract] OR "chromosome 10"[Title/Abstract] OR "maximal safe resection"[Title/Abstract] OR "extent of resection"[Title/Abstract] OR "radiotherapy"[Title/Abstract] OR "temozolomide"[Title/Abstract] OR "chemoradiotherapy"[Title/Abstract] OR "recurrent glioblastoma"[Title/Abstract]) AND ("Adult"[Mesh] OR adult*[Title/Abstract])

**2.2 Scopus**

TITLE-ABS-KEY(glioblastoma* OR "glioblastoma multiforme" OR "IDH-wildtype glioblastoma" OR "IDH wild-type glioblastoma") AND TITLE-ABS-KEY("IDH-wildtype" OR "IDH wild-type" OR IDH1 OR IDH2 OR "WHO CNS5" OR "molecular diagnosis" OR "molecular classification") AND TITLE-ABS-KEY(epidemiology OR incidence OR survival OR diagnosis OR biomarker* OR MGMT OR surgery OR resection OR radiotherapy OR temozolomide OR chemotherapy OR recurrence OR "supportive care" OR palliative OR "artificial intelligence" OR radiomics OR implementation OR access OR "health system*")

**3.3 Web of Science**

TS=(glioblastoma* OR "glioblastoma multiforme" OR "IDH-wildtype glioblastoma" OR "IDH wild-type glioblastoma") AND TS=("IDH-wildtype" OR "IDH wild-type" OR IDH1 OR IDH2 OR "WHO CNS5" OR "molecular diagnosis" OR "molecular classification") AND TS=(epidemiology OR incidence OR survival OR diagnosis OR biomarker* OR MGMT OR surgery OR resection OR radiotherapy OR temozolomide OR chemotherapy OR recurrence OR "supportive care" OR palliative OR "artificial intelligence" OR radiomics OR implementation OR access OR "health system*")

**3. Regional and implementation-focused searches**

3.1 **PubMed/MEDLINE**

("Glioblastoma"[Mesh] OR glioblastoma*[Title/Abstract] OR "IDH-wildtype glioblastoma"[Title/Abstract] OR "IDH wild-type glioblastoma"[Title/Abstract]) AND (Mexico[Title/Abstract] OR Mexican[Title/Abstract] OR "Latin America"[Title/Abstract] OR "Latin American"[Title/Abstract] OR Brazil[Title/Abstract] OR Brazilian[Title/Abstract] OR Argentina[Title/Abstract] OR Argentine[Title/Abstract] OR Chile[Title/Abstract] OR Colombia[Title/Abstract] OR Peru[Title/Abstract]) AND (epidemiolog*[Title/Abstract] OR incidence[Title/Abstract] OR survival[Title/Abstract] OR prognosis[Title/Abstract] OR "molecular diagnos*"[Title/Abstract] OR IDH1[Title/Abstract] OR IDH2[Title/Abstract] OR MGMT[Title/Abstract] OR surgery[Title/Abstract] OR resection[Title/Abstract] OR radiotherap*[Title/Abstract] OR temozolomide[Title/Abstract] OR access[Title/Abstract] OR disparity[Title/Abstract] OR disparities[Title/Abstract] OR implementation[Title/Abstract] OR "health system*"[Title/Abstract] OR registry[Title/Abstract] OR cost*[Title/Abstract])

**3.2 Scopus**

TITLE-ABS-KEY(glioblastoma* OR "IDH-wildtype glioblastoma" OR "IDH wild-type glioblastoma") AND TITLE-ABS-KEY(Mexico OR Mexican OR "Latin America" OR "Latin American" OR Brazil OR Brazilian OR Argentina OR Argentine OR Chile OR Colombia OR Peru) AND TITLE-ABS-KEY(epidemiology OR "molecular diagnosis" OR IDH1 OR IDH2 OR MGMT OR surgery OR radiotherapy OR temozolomide OR survival OR access OR disparities OR implementation OR "health system*" OR cost* OR registry)

**3.3 Web of Science**

TS=(glioblastoma* OR "IDH-wildtype glioblastoma" OR "IDH wild-type glioblastoma") AND TS=(Mexico OR Mexican OR "Latin America" OR "Latin American" OR Brazil OR Brazilian OR Argentina OR Argentine OR Chile OR Colombia OR Peru) AND TS=(epidemiology OR "molecular diagnosis" OR IDH1 OR IDH2 OR MGMT OR surgery OR radiotherapy OR temozolomide OR survival OR access OR disparities OR implementation OR "health system*" OR cost* OR registry)

**4. Treatment- and domain-specific search concepts**

| Domain | Main search concepts |
| --- | --- |
| Molecular diagnosis | IDH1, IDH2, MGMT, EGFR, TERT, +7/−10, WHO CNS5, molecular classification |
| Surgery | maximal safe resection, extent of resection, fluorescence-guided surgery, 5-ALA, intraoperative MRI, awake surgery |
| Radiotherapy | radiotherapy, chemoradiotherapy, hypofractionation, stereotactic radiotherapy, radiosurgery |
| Systemic treatment | temozolomide, chemotherapy, targeted therapy, immunotherapy |
| Recurrence | recurrent glioblastoma, salvage therapy, reoperation, reirradiation |
| Supportive care | corticosteroids, seizures, venous thromboembolism, rehabilitation, palliative care |
| Emerging strategies | tumor treating fields, viral therapy, cellular therapy, immunotherapy, blood-brain barrier, DNA damage response |
| AI/radiomics | artificial intelligence, machine learning, deep learning, radiomics, imaging biomarkers |
| Implementation | implementation, health systems, access, disparities, referral, capacity, resource-limited settings |

**5. Selection and prioritization of evidence**

Studies were prioritized according to their relevance to adult IDH-wildtype glioblastoma and contemporary clinical practice or implementation. Priority was given to randomized clinical trials, current clinical practice guidelines and consensus statements, prospective and registry-based studies, systematic reviews and meta-analyses, contemporary epidemiological studies, and regional studies addressing diagnosis, treatment delivery, outcomes, access, or health-system capacity. Early-phase and translational studies were considered when they provided relevant information on biological rationale, safety, feasibility, pharmacology, or preliminary activity, but were not considered practice-changing in the absence of confirmatory evidence. Studies with limited direct relevance to adult IDH-wildtype glioblastoma were used selectively for historical, biological, or contextual purposes.

**6. Identification and selection of regional evidence**

Regional evidence was specifically sought using geographic terms combined with disease-, diagnostic-, treatment-, and health-system-related concepts. Studies from Mexico were prioritized when available. Evidence from other Latin American countries was included when it provided contemporary information relevant to epidemiology, molecular diagnostics, treatment access or delivery, outcomes, resource constraints, or implementation. Because regional evidence was heterogeneous and frequently derived from tertiary or referral centers, such studies were interpreted as contextual evidence rather than as representative of the entire Latin American population.

**7. Methodological scope**

This search strategy was used to support a structured narrative review rather than a systematic review. Accordingly, duplicate independent screening, formal risk-of-bias assessment, predefined quantitative eligibility criteria, and meta-analysis were not performed. The final selection emphasized clinical relevance, methodological strength, contemporary applicability, and relevance to the Mexican and regional implementation context.
